# Medicaid Expansion Among Nonelderly Adults and Cardiovascular Disease: Efficiency Vs. Equity

**DOI:** 10.1111/1468-0009.70004

**Published:** 2025-03-21

**Authors:** LUKE E. BARRY, SANJAY BASU, MAY WANG, ROCH A. NIANOGO

**Affiliations:** ^1^ Fielding School of Public Health University of California Los Angeles; ^2^ Research and Development, Waymark San Francisco; ^3^ California Center for Population Research University of California Los Angeles

**Keywords:** Medicaid, disparities, cardiovascular diseases, cost‐effectiveness, economic evaluation, social policies

## Abstract

**Policy Points**

- Evidence suggests Medicaid expansion has improved cardiovascular disease (CVD) outcomes, especially among those of lower socioeconomic status. However, less is known about the cost‐effectiveness of Medicaid in achieving these outcomes and reducing CVD disparities.
- We found that Medicaid expansion resulted in a reduction in CVD incidence, suggesting that it was cost‐effective in reducing CVD outcomes and equity enhancing but with a high degree of uncertainty.
- Policymakers will need to trade‐off among a number of different factors in consideration of the value of Medicaid including health (especially in treating the chronically ill), financial protection, reduced uncompensated care, and health disparities.

**Context:**

Evidence suggests Medicaid expansion has improved cardiovascular disease (CVD) outcomes, especially among those of lower socioeconomic status. However, less is known about the cost‐effectiveness of Medicaid in achieving these outcomes and reducing CVD disparities. We use distributional cost‐effectiveness analysis methods to examine the efficiency and equitability of Medicaid expansion in reducing CVD outcomes.

**Methods:**

A Monte Carlo Markov‐chain microsimulation model was developed to examine lifetime changes in CVD outcomes and disparities as a result of expansion and the associated cost and quality‐of‐life impacts.

**Findings:**

Medicaid expansion was associated with a reduction of 11 myocardial infarctions, eight strokes, and four CVD deaths per 100,000 person‐years compared with no expansion. The largest reductions occurred for those with lower income and education, and those of Black and Hispanic race/ethnicity. We found that the benefits of expansion generally balanced out the costs while redistributing health from higher to lower income groups. In probabilistic sensitivity analysis, we found—using a health opportunity cost threshold of $150,000—that Medicaid expansion was cost‐effective in reducing CVD outcomes 53% of the time and both cost‐effective (efficient) and equity enhancing 26% to 29% of the time.

**Conclusions:**

Medicaid expansion resulted in a reduction in CVD incidence, suggesting that it was both cost‐effective and equity enhancing in reducing CVD outcomes but with a high degree of uncertainty.

In january 2014, at the discretion of individual states, the us patient Protection and Affordable Care Act (ACA) expanded Medicaid coverage to adults (aged 19–64 years) with family incomes below 138% of the federal poverty line (FPL). Evidence suggests this coverage has increased access and affordability of health care, especially for those of lower socioeconomic status (SES), while reducing uncompensated care and out‐of‐pocket (OOP) costs.^1^, ^2^, ^3^ As national policymakers consider whether to expand Medicaid coverage further, they must trade‐off among a number of factors, including health (especially in treating the chronically ill), financial protection, reduced uncompensated care, and health disparities.^1^

Cardiovascular diseases (CVDs) are among of the most common chronic conditions in the United States.^4^ They account for one‐third of deaths annually at substantial economic and humanistic cost and disproportionately affect those of lower SES.^5^, ^6^ By targeting Medicaid expansion toward lower income families, a priority of the expansion was to reduce socioeconomic as well as racial disparities in health.^7^ Recent evidence suggests Medicaid expansion has improved CVD outcomes and reduced CVD risk factors, especially among those of lower SES,^8^, ^9^, ^10^ in part through increased access to care and preventive therapies as a result of insurance coverage.^11^, ^12^, ^13^ It remains unclear whether Medicaid expansion can cost‐effectively reduce the burden of CVD and disparities therein. Although Medicaid expansion has been linked to health improvements other than CVDs, evaluating expansion in relation to a specific disease area helps to inform the debate on expanding coverage to individuals most likely to benefit to more efficiently allocate public resources.^1^, ^14^, ^15^, ^16^ Our analysis considers whether Medicaid expansion was cost‐effective/welfare enhancing in reducing CVD outcomes and whether social welfare was further enhanced via its redistributional nature.

Distributional cost‐effectiveness analysis (DCEA) methods can be used to address equity concerns that arise in assessments of health interventions. In particular, these concerns can be for reducing what may be considered unfair differences in health according to equity‐relevant subgroups, such as SES or race/ethnicity, while improving allocative efficiency/cost‐effectiveness.^17^ DCEA methods extend traditional cost‐effectiveness methods by analyzing distributions of health benefits and opportunity costs within the general population according to equity‐relevant subgroups and can explore the implications of prioritizing access to an intervention for recipients vs. nonrecipients.^18^ These analyses and implications are particularly relevant when examining the value of Medicaid's expansion to lower income adults. This paper uses a microsimulation state‐transition model to examine the distributional cost‐effectiveness of Medicaid expansion in reducing CVD outcomes across equity‐relevant subgroups (SES and race/ethnicity), which are a priority of Medicaid.

## Methods

### Model Overview

This model adheres to the Consolidated Health Economic Evaluation Reporting Standards 2022 for cost‐effectiveness analysis (CEA).^19^ We used a conservative cost‐effectiveness threshold (society's willingness to pay [WTP] for a quality adjusted life‐year [QALY]) of $150,000 to convert health opportunity costs into QALYs. This is the upper‐bound threshold recommended by the US Institute for Clinical and Economic Review.^20^ Results were presented as incremental net health benefit (INHB  =  [differences in effects] − [difference in costs / WTP]).^21^, ^22^ This study used publicly available deidentified aggregated data and, as such, did not qualify as human subject research; therefore, Institutional Review Board approval was not required. All analyses were conducted in R version 4.3.1.

**Figure 1:**
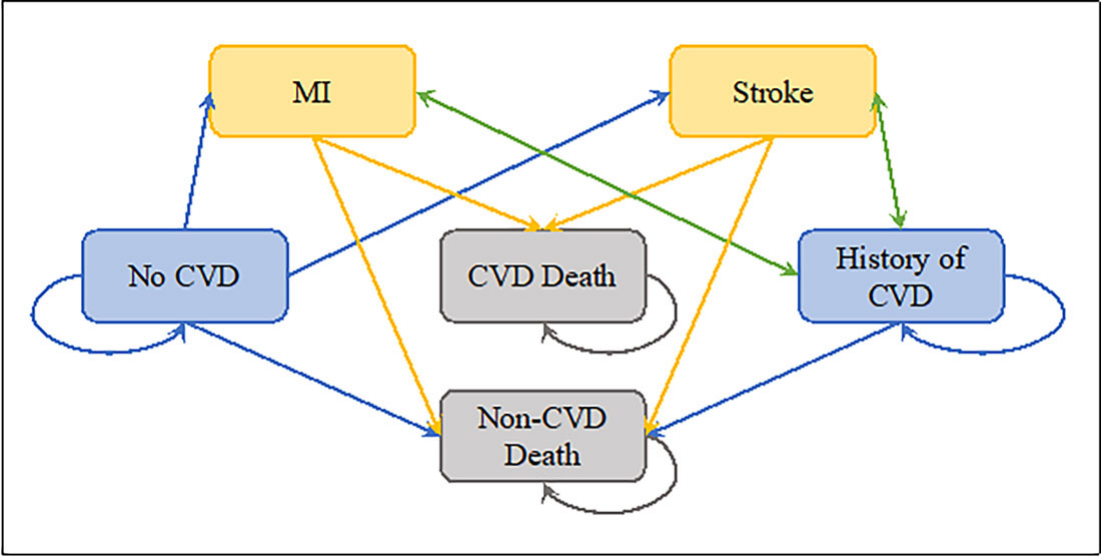
State‐Transition Diagram Outlining Possible Transitions for Each Individual in Each Cycle [Colour figure can be viewed at wileyonlinelibrary.com] Individuals begin in one of two states (“no CVD” or “history of CVD”). Each cycle an individual can die from other causes (“non‐CVD death”) or have one of two CVD events (“MI” or “stroke”), which can result in a “CVD death”. In each cycle the probability of transitioning to a given state is conditional on firstly not entering the “non‐CVD Death” state. CVD, cardiovascular disease; MI, myocardial infarction.

A Monte Carlo Markov‐chain microsimulation model was used to estimate and compare the costs and effects between two arms. A feature of DCEA is to consider both recipients and nonrecipients of the intervention.^18^ Hence each arm of our model simulated costs and effects for a nationally representative sample of all (noninstitutionalized) US adults aged 19 to 64 years, not just those eligible for Medicaid under expansion. The subgroup of Medicaid‐eligible individuals within this sample were those who were uninsured, aged 19 to 64 years, and had family incomes less than 138% of the FPL. Thus, we estimated and compared the costs and effects between the following groups: 1) a nationally representative sample of US adults aged 19 to 64 years, which includes a subgroup of Medicaid‐eligible individuals who do not receive Medicaid (nonexpansion); and 2) a nationally representative sample of US adults aged 19 to 64 years, which includes a subgroup of Medicaid‐eligible individuals who do receive Medicaid (expansion). All Medicaid‐eligible individuals in the expansion arm were assumed to receive the intervention effects. Four CVD outcomes were modeled (Figure 1), which have been linked to Medicaid expansion^8^, ^9^, ^10^: 1) nonfatal myocardial infarction (MI), 2) nonfatal stroke, 3) fatal MI, and 4) fatal stroke. Microsimulation allowed for heterogeneity across individuals’ demographic characteristics and CVD risk factors and disaggregation of results according to subgroups of the population as part of DCEA.

A limited societal perspective was applied, which includes health care costs and wider societal costs, namely lost productivity, but not other societal costs such as impacts on future consumption or caregiver burden. A span of lifetime horizon or until everyone turned 85 years (the age until which CVD and non‐CVD risks predictions were available) was modeled though all individuals switched to Medicare only at the age of 65 years. This means that Medicaid expansion has no direct impact after 65 years of age, except for the persistence of effects that occurred before 65 years of age (e.g., reduced systolic blood pressure at the age of 40 years can reduce the risk of a MI at the age of 70 years). Although individuals may transition in and out of Medicaid, owing to changes in income for example, we assumed that once an individual is enrolled in Medicaid they do not transition out of it (until 65 years of age). This is a closed model^23^ in that we simulate outcomes for an initial cross‐section of the US population with and without expansion. Each cycle of the model represents one year. Half‐cycle corrections were applied, and a discount rate of 3% was used.^20^

### Data

**Figure 2:**
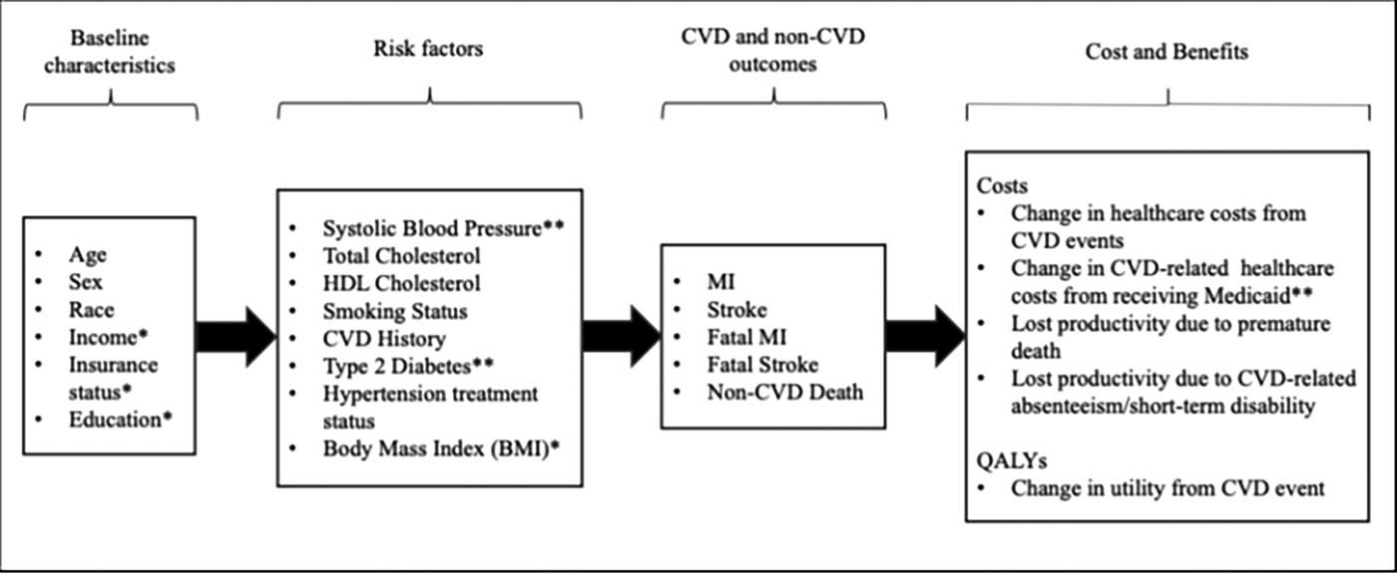
Parameters Used to Simulate Transitions Between States and Their Associated Costs and Benefits *These variables were used to predict costs and QALYs alongside other variables but were not used to predict CVD outcomes. **These variables were used to model changes as a result of Medicaid expansion: changes in systolic blood pressure and CVD‐related health care costs (routine primary care visits and prescriptions) were modeled directly, while changes in type 2 diabetes status were modeled via HbA_1c_. BMI, body mass index; CVD, cardiovascular disease; HbA_1c_, hemoglobin A1_c_; HDL, high‐density lipoprotein; MI, myocardial infarction; QALY, quality adjusted life‐year.

**Figure A1:**
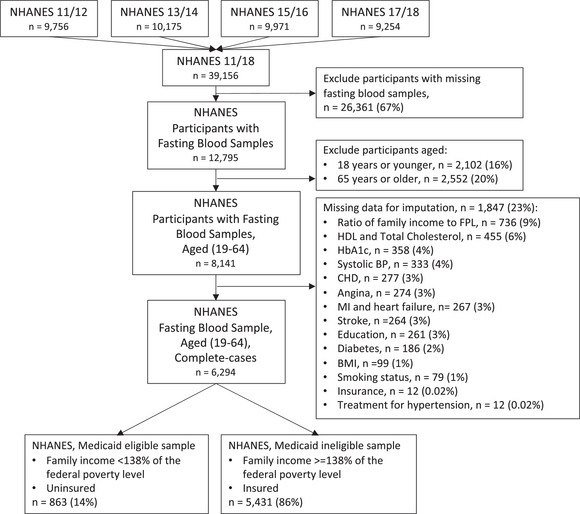
Flow Diagram Demonstrating the Data Selection Process and Variable Missingness for Multiple Imputation BMI, body mass index; BP, blood pressure; CHD, congenital heart disease; FPL, federal poverty line; HDL, high‐density lipoprotein; MI, myocardial infarction; NHANES, National Health and Nutrition Examination Survey.

A nationally representative sample of the civilian, noninstitutionalized population of American adults (≥18 years) from the National Health and Nutrition Examination Survey (NHANES; 2011–2018, *n*  =  39,156) was used to simulate individual outcomes. Our final sample included only individuals from the fasting blood sample who had measures of total and high‐density lipoprotein cholesterol and hemoglobin A_1c_ (HbA_1c_), which were used to predict CVD risks, and those aged 19 to 64 years (*n*  =  8,141; Figure A1 and Table A1). These years (2011–2018) were chosen so that the sample size was as large as possible while ensuring that the overall distribution of insurance approximately matched the distribution in 2013/2014 when Medicaid was expanded and that a consistent categorization of race/ethnicity was used. Prior to 2011, fewer categories were used. Fasting blood sample weights corrected for survey nonresponse and differential sampling and multiple imputation (10 imputed data sets with 20 iterations per data set using the miceRanger package in R^24^) were used to account for item nonresponse (2% missing data). We extracted additional input parameters—costs, utilities, and transition probabilities—from the literature, including measures of dispersion for probabilistic analysis. Transition probabilities, costs, and utilities were updated annually to reflect increasing age and changing CVD history. The pathways among variables are presented in Figure 2 and are described in brief below with additional detail available in the Appendix.

### Transition Probabilities

Annual risk of a MI or stroke was estimated using NHANES characteristics (Table A2) and the Framingham risk equation, adapted to differentiate risk beyond age and sex using the pooled cohort atherosclerotic CVD (ASCVD) risk equations.^25^, ^26^, ^27^ We used the Framingham equation because it differentiates among types of CVD event (fatal and nonfatal MI and stroke) and because of evidence that more recent equations used limited data, were overfitted, and may produce less accurate estimates of CVD risk.^28^, ^29^ The C statistics for the Framingham risk equation were 0.74 in men and 0.77 in women.^30^ Risks were weighted using the ASCVD equations^27^ to provide more granular predictions according to, for example, race, systolic blood pressure, and diabetes status. Annual risk of MI or stroke was multiplied by a factor of two for those with a history of CVD.^25^ Risk of dying from a MI or stroke was estimated using validated age‐ and sex‐specific equations to predict the incidence of fatal‐to‐total MI and fatal‐to‐total stroke.^25^, ^26^ Rate of non‐CVD death by age and sex was extracted from the Centers for Disease Control and Prevention WONDER database.^31^ The 2013 to 2014 age‐ and sex‐specific underlying cause of death rates for all‐causes were subtracted from death rates where the underlying cause was an MI (International Statistical Classification of Diseases, Tenth Revision [ICD‐10] codes I21–I22^32^) or a stroke (ICD‐10 codes I60–I69^33^). All estimates were converted to annual probabilities.^21^

### Intervention

The effect of expansion was modeled as a one‐time reduction in systolic blood pressure (mm Hg) and HbA_1c_ (%), which, in turn, influenced MI/stroke risk. This model was based on a study that used NHANES data (2005–2016) to estimate the change in cardiovascular risk factors among individuals aged 19 to 64 years with family incomes below 138% of the FPL between expansion and nonexpansion states, before and after the (state‐specific) expansion date.^10^ The authors examined cholesterol, blood pressure, and blood glucose, finding significant reductions after expansion for systolic blood pressure (−3.03 mm Hg; 95% CI, −5.33 to −0.73) and HbA_1c_ (−0.14 percentage points; 95% CI, −0.24 to −0.03).^10^ Other CVD benefits have been reported in relation to expansion,^13^ which include reduction in hospitalizations, morbidity and mortality, increases in routine care, screening, and prescriptions, as well as decreases in the rates of deferred care.^34^, ^35^, ^36^, ^37^, ^38^ To avoid the risk of double‐counting CVD benefits we modeled only the effect of expansion on CVD risk factors,^10^ which, in turn, affected the likelihood of a CVD event and CVD mortality.

### Costs and Utilities

Once an individual's CVD outcomes were simulated, corresponding health care costs and QALYs incurred by each individual were estimated, using their NHANES characteristics (Table A2), and a prediction equation examining the association of CVD with health care costs and utility.^39^ This equation used data from the Medical Expenditure Panel survey (2011–2016), which covers an extensive range of health care utilization events to estimate differences in the total cost of utilization and health utility (SF‐6D^40^) according to CVD diagnoses (including MI and stroke) while adjusting for confounders (see Appendix Methods). Because each cycle of our model was one year, these utility estimates were converted directly to QALYs—the outcome measure used in our CEA.

Health care costs were increased by 33% in the year of the MI or stroke.^39^ Lost productivity costs were included as the cost of workplace absenteeism and short‐term disability claims—incurred in the year of a MI or stroke^41^—and 100% lost earnings (2021 US average annual earnings^42^) from a premature death (CVD or non‐CVD) incurred every year before retirement (65 years of age). We also included the estimated cost of government administration per enrollee^43^, ^44^ apportioned to CVD using the proportion of Medicaid spending attributed to heart disease.^14^ In addition to costs associated with CVD events (MI or stroke), individuals receiving Medicaid also used more preventive services for CVD (see Appendix Methods).^13^ These costs were assumed to achieve the reduction in CVD biomarkers that we model. All costs were inflated to 2021 US dollars using the Personal Consumption Expenditures Inflation Indices from the Bureau of Economic Analysis.^45^

For DCEA, it is important to model the incidence of the increase in total health care costs following expansion (i.e., OOP vs. non‐OOP costs) as well as the total population, not just Medicaid recipients, so that distributional effects can be examined.^22^ The increase in total health care costs was categorized by payment source: OOP costs (paid by self or family), which decreased following expansion; and non‐OOP costs (paid by others; e.g., insurers), which increased such that the increase in non‐OOP costs was larger than the increase in total (OOP plus non‐OOP) health care costs.^46^ Medicaid premiums are generally not allowed for families with incomes less than 150% of the FPL, so OOP costs are the main source of expenditure for those receiving Medicaid.^2^ Thus, we model the intervention as a wealth transfer from higher to lower income groups such that, for individuals receiving Medicaid, their OOP costs are reduced while the increase in their total health care costs (plus government administration costs) are allocated evenly across individuals with family incomes greater than 150% of the FPL. Importantly, this assumes that the incidence of baseline costs are the same between expansion and nonexpansion arms. This is a simplifying assumption and may differ depending on the level and efficiency by which costs for uninsured individuals in the nonexpansion arm, for example uncompensated hospital care, are compensated by public funds.^47^

### Simulation

A random sample of adults, aged 19 to 64 years, was selected with replacement from each of ten imputed data sets (each *n*  =  8,141) with 2,000 bootstrapped replications per data set (20,000 simulations in total). To reduce unnecessary variation, Monte Carlo sampling of the data sets and input parameters was the same for each arm. Results were averaged and Monte Carlo standard errors estimated across replications^48^ in total and for each equity‐relevant subgroup: family income, highest educational achievement, and race/ethnicity. This was done for each imputed data set before combining estimates across imputations using Rubin's rules.^49^ We estimated the incidence rate of stroke and MI per 100,000 person‐years (excluding deaths) and assessed face‐validity by comparing age‐specific incidence rates of MI and stroke with external sources (Appendix Results).

### Distributional Analysis

For DCEA, we examined the efficiency/cost‐effectiveness and its distribution according to equity‐relevant subgroups, Medicaid‐eligible individuals only, and those with a history of CVD at baseline. For each equity‐relevant subgroup, we used the Atkinson social welfare function^50^ to calculate the equally distributed equivalent of health (EDEH) using a conservative inequality aversion parameter (ε; how society weights health transfers from the better‐ to the worse‐off) of 0.5,^51^ with sensitivity analysis across values of inequality aversion (ε). The EDEH can be thought of as the equity‐adjusted net health benefit (NHB) of each arm.^22^ We converted this to Atkinsons index of inequality (Aε  =  1 − [EDEH / NHB]), which ranges from zero to one and reflects the inequality burden in each arm. A value of zero suggests either no inequality because the NHB is equal to the EDEH regardless of inequality aversion (ε) or a society with no aversion to inequality (ε  =  0, thus EDEH  =  NHB). This was used to estimate the burden of inequality in each arm (Aε × NHB) before estimating the change in the burden of health inequality as a result of Medicaid expansion in population terms^22^, ^52^ using NHANES 2013 to 2014 weighted population estimates (*N*  =  189,980,531). Results were presented on an efficiency‐equity plane, which plots the total welfare gain or loss through efficiency or cost‐effectiveness (INHB) on the *y*‐axis and the excess welfare gain or loss through the intervention's redistributional effects on the *x*‐axis.

### Sensitivity Analysis

One‐way sensitivity analysis was conducted by sequentially varying the mean values of each parameter (excluding those that affect transition probabilities) by ±20% and examining the corresponding variation in the INHB using a tornado diagram. We conducted a probabilistic sensitivity analysis (PSA) examining the probability of expansion being cost‐effective (INHB > 0) relative to nonexpansion across WTP values ($0–$1,000,000) and presented results using a cost‐effectiveness acceptability curve. We repeated this, examining a shorter time horizon of ten years (Figure A5) and for the time period individuals were in receipt of Medicaid (i.e., restricting the time horizon to 65 years of age) (Figure A6).

Sensitivity of the incremental EDEH (IEDEH) was examined across WTP and inequality aversion (ε) values for each equity‐relevant group. We selected a conservative inequality aversion value (ε  =  0.5) because there is uncertainty as to the appropriate value, which may differ, for example, for aversion to pure health inequalities vs. socioeconomic inequalities in health.^22^, ^53^ As such, we also estimate the value of inequality aversion (ε) at which expansion is considered welfare enhancing (cost‐effective). That is, if expansion is found to reduce social welfare (not cost‐effective), what level of societal aversion to inequality would be required for its redistribution potential to offset this welfare reduction? Finally, we also conduct a DCEA‐PSA, examining the probability of expansion being both efficient and equity enhancing at the WTP value of $150,000 and inequality aversion value of 0.5.

## Results

### Effectiveness

Medicaid expansion was associated with a reduction of 11 MIs, eight strokes, and four CVD deaths per 100,000 person‐years compared with no expansion. The largest reductions occurred for those with lower income and education, and those of Black and Hispanic race/ethnicity (Tables A3). Incidence rates of MI and stroke were consistent with published estimates in total and across age‐groups (Table A4). When examining Medicaid‐eligible individuals only, expansion was associated with a reduction of 105 MIs, 77 strokes, and 34 CVD deaths per 100,000 person‐years (Table A3).

### Cost‐Effectiveness

Total costs and QALYs for the base‐case scenario (societal perspective, total population aged 19–64 years, lifetime horizon or until 85 years of age, probabilistic analysis) are presented overall and across equity‐relevant subgroups (Table 1 and Table A5). Total costs per person in the nonexpansion arm were $270,525, and the QALYs were 15.691. Total costs per person in the expansion arm were $270,551, and the QALYs were 15.694. Consequently, the INHB was positive (0.0031) at the WTP value of $150,000, indicating that Medicaid expansion was cost‐effective in reducing CVD outcomes but with a high degree of uncertainty (see Sensitivity Analysis below). In short, the overall increase in health care costs per individual receiving Medicaid plus the cost of administering the program per enrollee generally cancel out the health benefits and improved productivity.

**Table 1:** Incremental Costs, QALYs, Cost‐Effectiveness (INHB), and Equity‐Adjusted Cost‐Effectiveness (IEDEH) Per Person in Total and Across Subgroups Using a Cost‐Effectiveness WTP of $150,000 Per QALY

	Incremental Costs (2021), $	Incremental Effects (QALYs)	INHB (QALYs)	IEDEH (QALYs), ε = 0.5	IIEDEH (QALYs), ε = 1.5	EDEH (QALYs), ε = 2.5	Population Fraction
Total	−26	0.0029	0.0031	—	—	—	1
Medicaid eligible only	−2,146	0.028	0.0423	—	—	—	0.1
History of CVD	−665	0.0081	0.0125	—	—	—	0.05
Family income							
High	283	0.0001	−0.0017	0.0031	0.0031	0.0032	0.34
Medium	294	0.0001	−0.0019				0.28
Low	−308	0.0038	0.0059				0.15
Near poor	−544	0.0062	0.0099				0.06
Poor	−701	0.0106	0.0153				0.18
Education							
Master/Doctorate	257	−0.0002	−0.0019	0.0031	0.0033	0.0034	0.31
Associate/Bachelor	104	0.001	0.0003				0.33
GED/HS	−284	0.0073	0.0092				0.22
No degree	−526	0.007	0.0105				0.14
Race/ethnicity							
Asian	195	0.0015	0.0002	0.0031	0.0031	0.0031	0.06
Black	−234	0.0021	0.0037				0.12
Hispanic	−140	0.0037	0.0046				0.17
Other race	101	0.0015	0.0008				0.04
White	19	0.0031	0.0029				0.61

CVD, cardiovascular disease; EDEH, equally distributed equivalent of health; GED, general education development; HS, high school; IEDEH, incremental equally distributed equivalent of health; INHB, incremental net health benefit; NHANES, National Health and Nutrition Examination Survey; QALY, quality adjusted life‐year; WTP, willingness to pay. The weighted NHANES population size in 2013/2014 was estimated to be 189,980,531. IEDEH results are presented across values of inequality aversion (ε) and equity‐relevant subgroups. These can be compared against the INHB for the full sample (0.0031). Where EDEH is great than INHB, this suggests that equity is improved according to the equity‐relevant subgroup as a result of expansion. Where the IEDEH or INHB are greater than zero, Medicaid expansion is welfare enhancing.

### Distributional Cost‐Effectiveness

**Figure 3:**
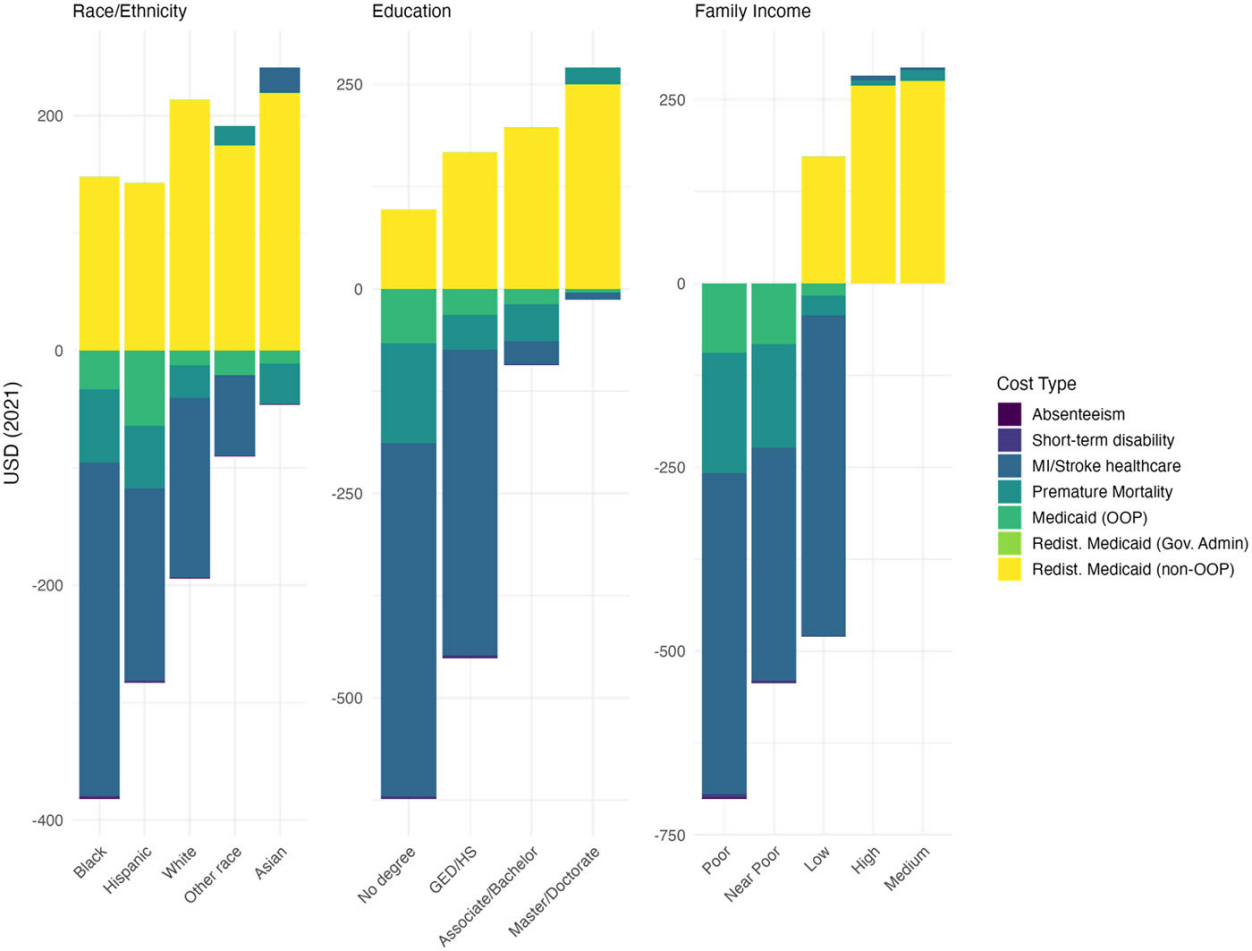
Incremental Costs (USD 2021) Per Person for the Base‐Case Scenario Across Equity‐Relevant Groups [Colour figure can be viewed at wileyonlinelibrary.com] GED, general educational development; HS, high school; MI, myocardial infarction; OOP, out of pocket; USD, United States dollars.

The INHB was highest for those with less education and lower income (Table 1) and, in particular, among those with a history of CVD at baseline and Medicaid‐eligible individuals (aged 19–64 years, uninsured and family income <138% FPL). This is driven, in part, by the redistributional effects of Medicaid expansion because the have higher QALYs (Table 1), lower OOP costs, fewer MI or strokes, and lower lost productivity owing to premature mortality, while the non‐OOP and government administration costs are spread across those in higher income groups (Figure 3).

**Figure 4:**
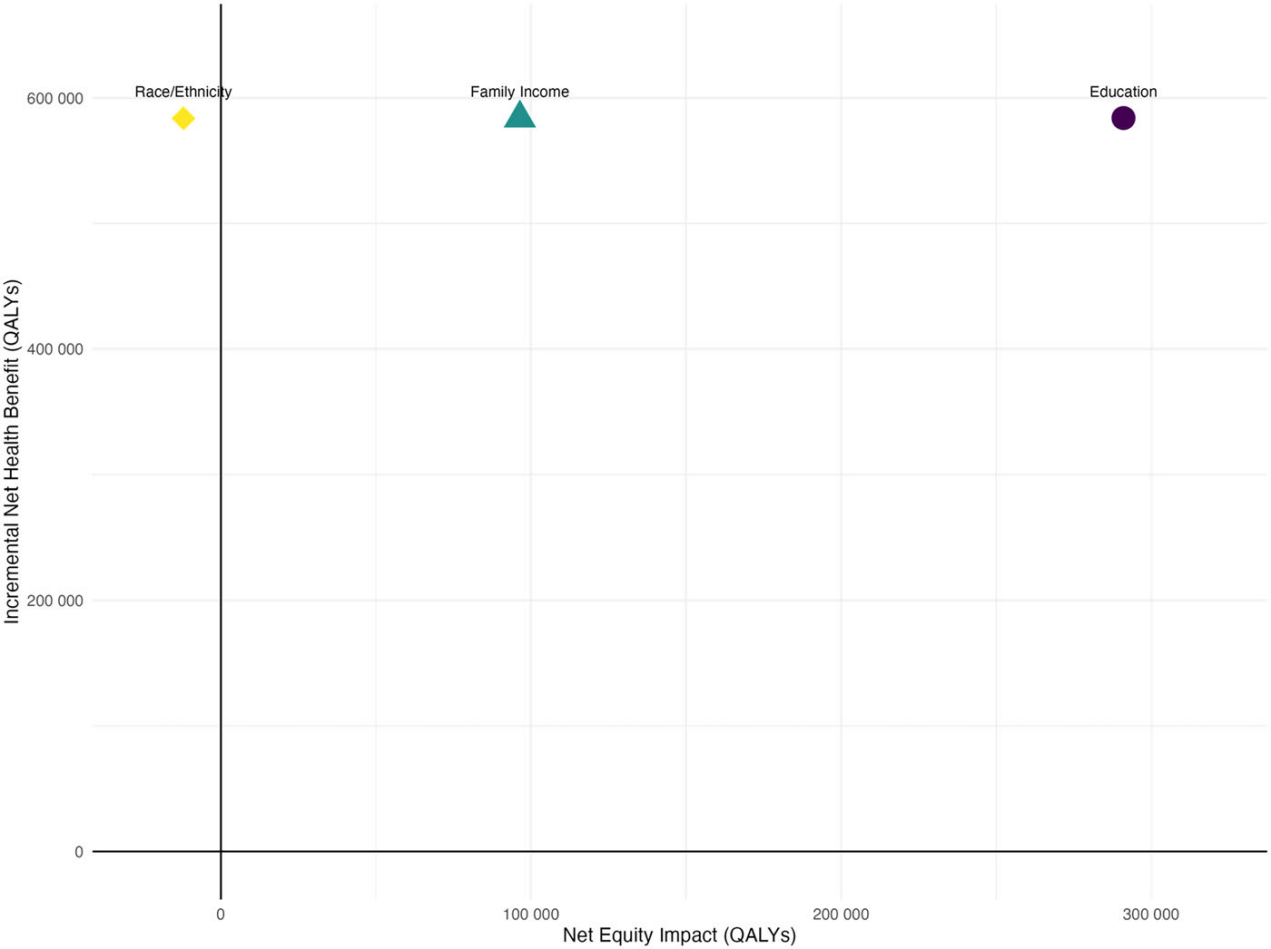
Equity‐Efficiency Plane of Medicaid Expansion Vs. No Medicaid Expansion for Equity‐Relevant Groups [Colour figure can be viewed at wileyonlinelibrary.com] Efficiency is measured as the INHB, using a cost‐effectiveness threshold of $100,000 (WTP), multiplied by the population (NHANES 2013/2014 population aged 19–64   =  189,980,531), and equity is measured as the net equity impact relative to the INHB, using inequality aversion (ε) parameter of 0.5 (see Distributional Analysis section for details). Results in the bottom‐right quadrant suggest Medicaid expansion is not efficient/cost‐effective (INHB < 0) but is equity enhancing (IEDEH > INHB). EDEH, equally distributed equivalent of health; INHB, incremental net health benefit; NHANES, National Health and Nutrition Examination Survey; QALY, quality adjusted life‐year; WTP, willingness to pay.

**Figure A2:**
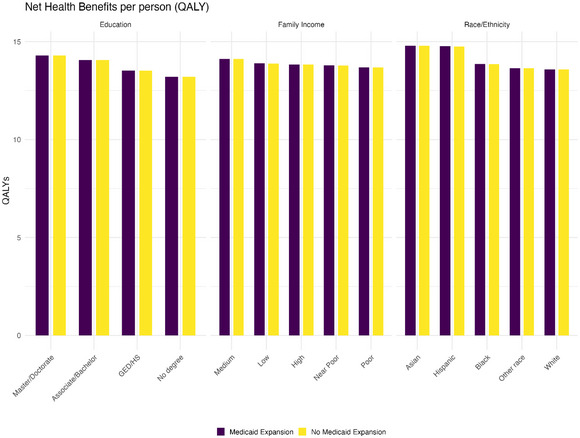
NHB (WTP  =  $150,000) Per Person for the Base‐Case Scenario Across Equity‐Relevant Groups Between Medicaid Expansion and No Expansion [Colour figure can be viewed at wileyonlinelibrary.com] See Tables 1 and A5 for detailed estimates of the differences between Medicaid expansion and no expansion. GED, general education development; HS, high school; NHB, net health benefit; QALY, quality adjusted life‐year; WTP, willingness to pay.

When adjusting the INHB to account for the distributional effects of expansion (IEDEH), equity was improved across income and educational levels but not race/ethnicity groups (Figure 4). Although the benefits of expansion in terms of CVD incidence occurred for those of Black and Hispanic race/ethnicity, this did not necessarily translate to improved equity because these groups did not have the lowest net health (effects − [costs / WTP]) in the nonexpansion arm (Figure A2).

### Sensitivity Analysis

**Figure A3:**
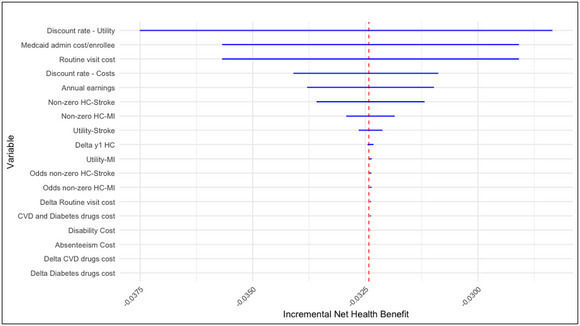
Deterministic Sensitivity Analysis Sequentially Varying Each Parameter in the Model by ±20% to Examine the Effect on the INHB [Colour figure can be viewed at wileyonlinelibrary.com] This scenario relates to the base‐case in our analysis but uses a deterministic analysis using a single simulation from one imputed data set of 8,141 individuals. As a result, the INHB may differ from the results presented in the main manuscript. This figure highlights the relative importance of each parameter in terms of its impact on cost‐effectiveness. It was not possible to examine variables that affect transition probabilities—change in systolic blood pressure, change in HbA_1c_, CVD history risk multiplier—in deterministic sensitivity analysis. CVD, cardiovascular disease; HbA_1c_, hemoglobin A_1c_; HC, health care costs; INHB, incremental net health benefit; MI, myocardial Infarction; NHB, net health benefit; y1, year one.

**Figure A4:**
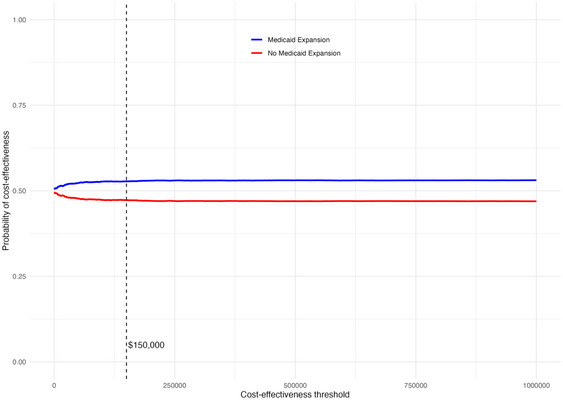
PSA For the Base‐Case scenario (Lifetime Horizon or Age 85) Across WTP values [Colour figure can be viewed at wileyonlinelibrary.com] PSA, probabilistic sensitivity analysis; WTP, willingness to pay.

**Figure A5:**
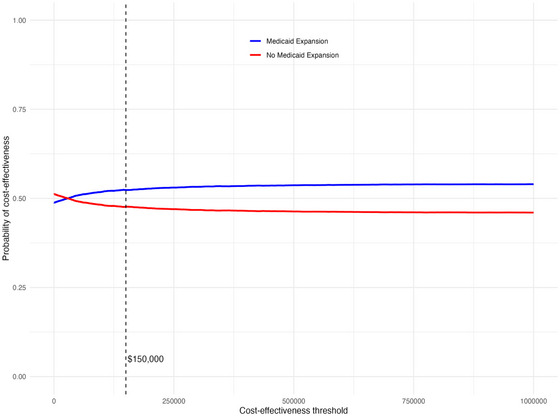
PSA for 10‐Year Time Horizon scenario Across WTP Values [Colour figure can be viewed at wileyonlinelibrary.com] PSA, probabilistic sensitivity analysis; WTP, willingness to pay.

**Figure A6:**
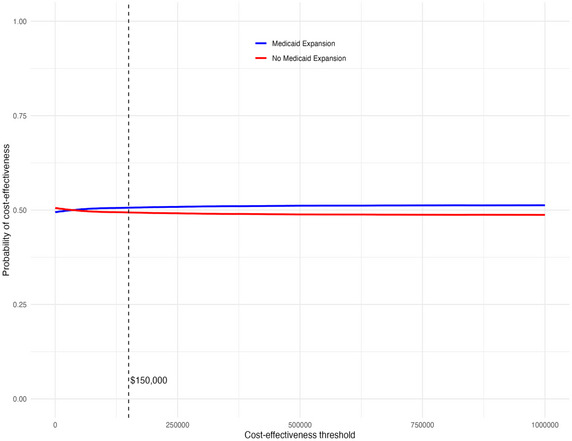
PSA for Lifetime Horizon or Age 65 Across WTP Values [Colour figure can be viewed at wileyonlinelibrary.com] PSA, probabilistic sensitivity analysis; WTP, willingness to pay.

The most influential variables in the deterministic sensitivity analysis (Figure A3) were the discount rates, Medicaid administration costs, cost of a routine visit, and average annual earnings. For the PSA, at the WTP value of $150,000, expansion was cost‐effective 53% of the time (Figure A4). A shorter time horizon (10 years) reduced the INHB, as did lowering the age by which individuals could be in the model (85 to 65 years old). Essentially, the health benefits of Medicaid persist beyond the eligibility window and are more valuable in older age‐groups when CVD risks are higher (Figures A4, A5, A6).

**Figure A7:**
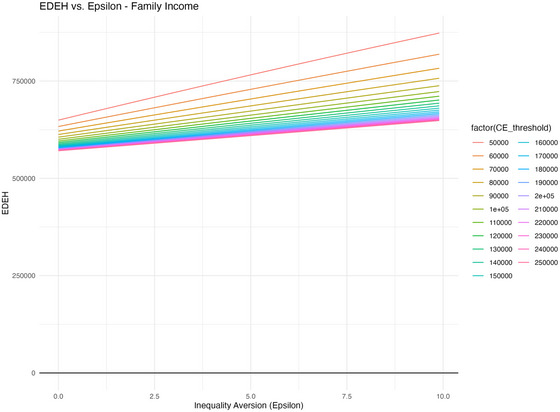
Sensitivity Analysis Examining Change in Net EDEH Between Arms Across Values of WTP ($50,000‐$250,000) and Inequality Aversion Parameters (ε: 0–10) for Family Income [Colour figure can be viewed at wileyonlinelibrary.com] The vertical axis (where ε  =  0) reduced to the INHB. CE, cost‐effectiveness; EDEH, equally distributed equivalent of health; INHB, incremental net health benefit; WTP, willingness to pay.

**Figure A8:**
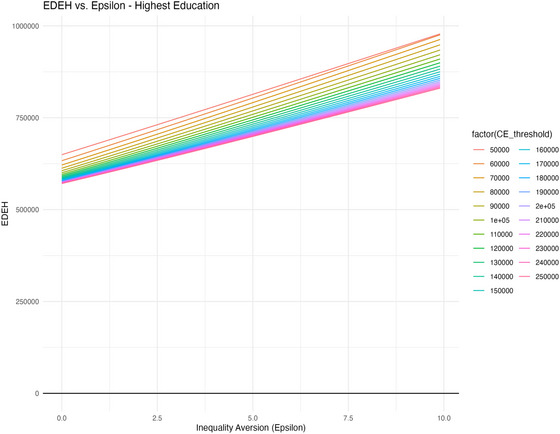
Sensitivity Analysis Examining Change in Net EDEH Between Arms Across Values of WTP ($50,000‐$250,000) and Inequality Aversion Parameters (0‐10) for Education [Colour figure can be viewed at wileyonlinelibrary.com] CE, cost‐effectiveness; EDEH, equally distributed equivalent of health; WTP, willingness to pay.

**Figure A9:**
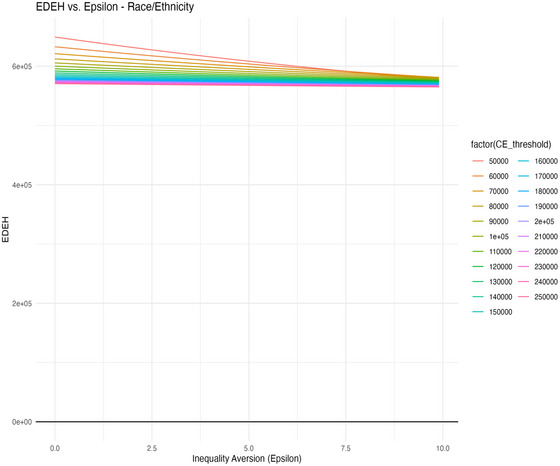
Sensitivity Analysis Examining Change in Net EDEH Between Arms Across Values of WTP ($50,000‐$250,000) and Inequality Aversion Parameters (0‐10) for Race/Ethnicity [Colour figure can be viewed at wileyonlinelibrary.com] CE, cost‐effectiveness; EDEH, equally distributed equivalent of health; WTP, willingness to pay.

The sensitivity of the IEDEH (equity‐adjusted INHB) for the base‐case scenario to variation in WTP and inequality aversion (ε) values are presented in Figures A7, A8, A9. For income and education, IEDEH is higher as inequality aversion (ε) increases across WTP values because those with lower income and education had lower net health in the nonexpansion arm, and NHBs from expansion were accrued by these groups. Although expansion was marginally welfare enhancing using a WTP value of $150,000, this welfare could be enhanced further at higher levels of societal inequality aversion (ε) for income and for education (Figure A7 and A8). For race/ethnicity, WTP values are less important at higher values of inequality aversion (ε) (Figure A9), highlighting the importance of cost redistributions as part of Medicaid expansion. In DCEA‐PSA, we found that, at the WTP value of $150,000 and inequality aversion (ε) of 0.5, the probability that expansion was both cost‐effective in reducing CVD outcomes and equity enhancing (that is the proportion of replications in the top right quadrant in Figure 4) was 29%, 28%, and 26% for education, family income, and race/ethnicity, respectively.

## Discussion

The purpose of this study was to examine the distributional cost‐effectiveness of Medicaid expansion in reducing CVD outcomes. We found that at the population level (aged 19–64 years), Medicaid expansion was associated with a reduction of 11 MIs, eight strokes, and four CVD deaths per 100,000 person‐years. The largest reductions occurred for those of lower income and education and those of Black and Hispanic race/ethnicity. In relation to its cost‐effectiveness, the cost of increased health care utilization plus the cost of administering Medicaid generally canceled out the health benefits (reduced fatal and nonfatal MIs and strokes) and avoided lost productivity when averaged across all nonelderly adults. Expansion was associated with a reduction in net health disparities across family income and education groups compared with a nonexpansion arm but not race/ethnicity. Importantly, net health accounts for the differences in health between expansion and nonexpansion arms as well as the health foregone in order to achieve these benefits (health opportunity costs). However, there was a high degree of uncertainty as to the cost‐effectiveness and equitability of Medicaid expansion in reducing CVD outcomes.

Although it can be unclear as to how financial protection should be weighed alongside traditional CEA outcomes, many DCEAs consider it as a separate outcome.^54^ Here, we model the financial protection offered by Medicaid directly in terms of reduced OOP costs so that it is implicit in our cost‐effectiveness results (INHB). Kaushal and Muchomba^46^ found that although OOP costs decreased following expansion, total health care costs increased as individuals receiving Medicaid increased health care utilization. Thus, non‐OOP costs increased by an even larger amount than total health care costs (increased total health care costs plus reduced OOP costs). We model this increased cost to capture the increased health care utilization from receiving health insurance and model the health benefits from this additional health care as reductions in systolic blood pressure and HbA_1c_,^10^ which, in turn, reduce CVD risks. Although the benefits of expansion—better health and reduced OOP costs—accrue to the recipient, the distribution of costs—increased non‐OOP and government administration costs—are more complex.

Medicaid expansion has been shown to reduce uncompensated care costs for health care providers, an aim of the ACA.^55^, ^56^ These providers incur significant costs in treating the uninsured who are often unable to pay these costs, leaving providers—in particular hospitals that serve vulnerable populations—with bad debt.^55^, ^56^ We assume the change in non‐OOP costs in the expansion arm is covered by Medicaid, which replaces medical spending that would primarily occur using private dollars, whether OOP or uncompensated care, with tax‐supported insurance coverage.^57^ However, uncompensated care costs in the nonexpansion arm may, in part, be financed through a complex web of public financial streams.^47^ Although we show that expansion is cost‐effective among those receiving it by reducing OOP costs and improving health, the degree of cost‐effectiveness will also depend on the level and efficiency by which non‐OOP costs are compensated by public funds in the nonexpansion arm.

Previous research has also considered the redistributive nature of Medicaid using tax payer funds when examining its value.^57^, ^58^ Sommers^57^ shows that the cost of each prevented death from Medicaid (2007, $327,000–$867,000) is far below the value of a statistical life (>$1 million), suggesting cost‐effectiveness even when accounting for the redistribution from private to public funds. Borgschulte and Vogler^59^ also consider the mortality benefits of Medicaid expansion and find the annual welfare gains to range from $21 to $102 billion, suggesting it would likely be cost‐effective when compared with the portion of annual Medicaid expenditure in 2017 ($70 billion) that is not a wealth transfer from public to private parties (∼$28 billion). As such, this conclusion is very dependent on the assumed proportion that is a wealth transfer (60%). Finkelstein and colleagues^58^ also show that Medicaid's value is derived by the reduction in transfers to other parties: for example, uncompensated care, through government intervention and from the provision of care to individuals whom might not have attained insurance without it.^57^, ^58^ They estimate this value to be between $0.4 and $1.2 per dollar spent on the program while noting this will partially depend on societal WTP, given Medicaid's redistributive nature, which may be considerably higher than a recipient's WTP.^58^ Here we model this redistribution and weight it using the Atkinson social welfare function and a conservative inequality aversion parameter (ε  =  0.5). We similarly find that whether expansion is cost‐effective in reducing CVD outcomes and equity enhancing is subject to a number of important assumptions regarding this redistribution.

These studies have focused on both CVD and non‐CVD mortality,^57^, ^59^ while one considered quality‐of‐life by mapping a course measure of well‐being to utility.^58^ We focus only on CVD, considering both mortality and more granular quality‐of‐life effects and similarly find that although Medicaid expansion was likely cost‐effective—particularly among those with a history of CVD at baseline—there was high uncertainty in this estimate. This was influenced by factors like value of a QALY and/or inequality aversion parameter (ε). It is possible that the WTP for a QALY may differ for different individuals: for example, according to income. This difference may also influence our results and should be an area of future research. When examining only those eligible for Medicaid, we found that individuals receiving Medicaid have lower OOP costs and better health, while the cost of their increased health care utilization (non‐OOP) is redistributed among those in higher income groups. Medicaid expansion comes with a substantial price tag: insured individuals use more health care that the uninsured; however, the welfare effects of expansion (from improved health and/or equity) may justify expansion, especially when targeted at those most likely to benefit.^14^, ^15^, ^16^ Policymakers will need to trade‐off among a number of different factors in considering the value of Medicaid, including health (especially in managing the chronically ill), financial protection, reduced uncompensated care, and health disparities.^1^

Our study is subject to limitations. First, we conducted our analysis using a national database, but results may differ across states. For example, although premiums are usually very small, in some states these premiums may be paid by Medicaid recipients.^2^ Secondly, we examine the value of Medicaid expansion in relation to CVD only; however; benefits beyond CVD have been reported. As discussed, this helps to inform the debate about expanding coverage to those who may benefit most (e.g., those at greater risk of CVD events owing to social and economic factors), but it also avoids risks of double‐counting benefits (e.g., mortality reductions will, in part, be owed to improved CVD health).^60^ Third, we estimated the impact of a one‐time legislative change over individuals’ lifetime, assuming its effect is constant over the lifetime and there are neither future changes that would impact Medicaid access nor do individuals switch in and out of Medicaid. Fourth, the effects of Medicaid expansion on the increased health care costs (e.g., 113% increase in total annual costs) and government administration costs remain constant in our model, which might overestimate the lifetime additional costs associated with expansion. Finally, many of the parameters in this model came from studies examining statewide effects often averaged across individuals who did not receive Medicaid and may, thus, underestimate the true effect per person of receiving the intervention.

## Conclusions

We found that Medicaid expansion resulted in a reduction in CVD incidence. Although we found that it was likely cost‐effective and equity enhancing in achieving these CVD outcomes, similar to previous studies of the cost‐effectiveness of Medicaid expansion, there was very high uncertainty as to these conclusions. Ultimately, policymakers will need to trade‐off among a number of important factors in considering the value of Medicaid, and we present a framework and results for assessing this value and quantifying its uncertainty in relation to CVD.

## Funding/Support

This study was supported by the National Institute on Minority Health and Health Disparities (NIMHD) [1K01MD014163], National Institutes of Health, Bethesda, MD. This research has been funded in part by training program T15LM013976 (Luke Barry) from the National Institutes of Health/National Library of Medicine. The funder had no role in study design, data collection and analysis, decision to publish, or preparation of the manuscript.

## Conflict of Interest Disclosures

All authors report no conflicts of interest.

## Methods

### Systolic Blood Pressure

We took the average of the first three readings. Where one of the first three readings was missing we calculated the average including the fourth reading. Where two or more readings were missing, we took the average of the remaining readings (https://wwwn.cdc.gov/nchs/nhanes/1999‐2000/BPX.htm). Individuals receiving Medicaid receive a one‐time reduction in systolic blood pressure.^10^

### Diabetes status

NHANES provides data on a number of diabetes related measures, such as self‐report, fasting blood glucose and Glycohemoglobin (%). Data on the effect of Medicaid expansion on diabetes related measures were available for HbA_1c_, thus we used the NHANES measure of Glycohemoglobin (%) to classify individuals as having diagnosed (doctor diagnosed self‐report) or undiagnosed diabetes (HbA_1c_ > 6.5% and no doctor diagnosed self‐reported diabetes).^61^ Individuals receiving Medicaid receive a one‐time reduction in HbA_1c_ as a result of Medicaid expansion.^10^ We assume the effect of Medicaid expansion on HbA_1c_ applied to individuals without doctor diagnosed diabetes.

**Table A1:** Baseline Characteristics and Proportion of Missing Data Across Waves From NHANES 2011 to 2018

Variable/Wave	2011/12, *N* = 2,086	2013/14, *N* = 2,129	2015/16, *N* = 1,996	2017/18, *N* = 1,930
Age in years				
Mean (SD)	41 (14)	42 (13)	42 (13)	43 (14)
*N* missing (% missing)	0 (0)	0 (0)	0 (0)	0 (0)
Stroke, *n* (%)				
No	1,966 (94)	2,015 (95)	1,899 (95)	1,821 (94)
Yes	47 (2.3)	39 (1.8)	37 (1.9)	53 (2.7)
*N* missing	73 (3.5)	75 (3.5)	60 (3.0)	56 (2.9)
MI, *n* (%)				
No	1,979 (95)	2,012 (95)	1,881 (94)	1,820 (94)
Yes	34 (1.6)	41 (1.9)	55 (2.8)	52 (2.7)
*N* missing	73 (3.5)	76 (3.6)	60 (3.0)	58 (3.0)
Coronary heart disease, *n* (%)				
No	1,981 (95)	2,018 (95)	1,895 (95)	1,835 (95)
Yes	25 (1.2)	34 (1.6)	38 (1.9)	38 (2.0)
*N* missing	80 (3.8)	77 (3.6)	63 (3.2)	57 (3.0)
Congestive heart failure, *n* (%)				
No	1,978 (95)	2,020 (95)	1,893 (95)	1,846 (96)
Yes	36 (1.7)	34 (1.6)	39 (2.0)	28 (1.5)
*N* missing	72 (3.5)	75 (3.5)	64 (3.2)	56 (2.9)
Angina pectoris, *n* (%)				
No	1,991 (95)	2,027 (95)	1,904 (95)	1,830 (95)
Yes	20 (1.0)	25 (1.2)	31 (1.6)	39 (2.0)
*N* missing	75 (3.6)	77 (3.6)	61 (3.1)	61 (3.2)
HDL cholesterol (mg/dL)				
Mean (SD)	53 (15)	53 (16)	55 (17)	53 (16)
*N* missing (% missing)	133 (6.4)	97 (4.6)	120 (6.0)	105 (5.4)
Total cholesterol (mg/dL)				
Mean (SD)	193 (41)	189 (41)	191 (42)	187 (40)
*N* missing (% missing)	133 (6.4)	97 (4.6)	120 (6.0)	105 (5.4)
Currently taking hypertension medication, *n* (%)				
No	1,696 (81)	1,732 (81)	1,627 (82)	1,542 (80)
Yes	386 (19)	395 (19)	367 (18)	384 (20)
*N* missing	4 (0.2)	2 (<0.1)	2 (0.1)	4 (0.2)
Systolic blood pressure (mm Hg)				
Mean (SD)	119 (16)	120 (16)	122 (17)	122 (18)
*N* missing (% missing)	100 (4.8)	75 (3.5)	58 (2.9)	100 (5.2)
Gender, *n* (%)				
Male	1,036 (50)	1,011 (47)	967 (48)	914 (47)
Female	1,050 (50)	1,118 (53)	1,029 (52)	1,016 (53)
*N* missing	0 (0)	0 (0)	0 (0)	0 (0)
Insurance status, *n* (%)				
Uninsured	597 (29)	561 (26)	455 (23)	393 (20)
Private	1,038 (50)	1,124 (53)	973 (49)	934 (48)
Medicare	69 (3.3)	54 (2.5)	76 (3.8)	99 (5.1)
Medicaid	214 (10)	225 (11)	211 (11)	297 (15)
Other plan	168 (8.1)	163 (7.7)	276 (14)	202 (10)
*N* missing	0 (0)	2 (<0.1)	5 (0.3)	5 (0.3)
Medicaid eligibility under expansion, *n* (%)				
Eligible	322 (15)	309 (15)	239 (12)	161 (8.3)
Ineligible	1,725 (83)	1,770 (83)	1,705 (85)	1,690 (88)
*N* missing	39 (1.9)	50 (2.3)	52 (2.6)	79 (4.1)
Highest education attained, *n* (%)				
No degree	423 (20)	421 (20)	416 (21)	361 (19)
GED/HS	410 (20)	431 (20)	422 (21)	441 (23)
Associate/Bachelor	619 (30)	658 (31)	573 (29)	629 (33)
Master/Doctorate	563 (27)	542 (25)	526 (26)	445 (23)
*N* missing	71 (3.4)	77 (3.6)	59 (3.0)	54 (2.8)
Race/ethnicity, *n* (%)				
Hispanic	456 (22)	496 (23)	615 (31)	507 (26)
White	713 (34)	857 (40)	584 (29)	558 (29)
Black	523 (25)	439 (21)	458 (23)	459 (24)
Asian	330 (16)	276 (13)	263 (13)	291 (15)
Other race	64 (3.1)	61 (2.9)	76 (3.8)	115 (6.0)
*N* missing	0 (0)	0 (0)	0 (0)	0 (0)
Current smoker, *n* (%)				
No	1,552 (74)	1,621 (76)	1,540 (77)	1,537 (80)
Yes	459 (22)	508 (24)	452 (23)	393 (20)
*N* missing	75 (3.6)	0 (0)	4 (0.2)	0 (0)
Family income, *n* (%)				
Poor	533 (26)	522 (25)	443 (22)	362 (19)
Near poor	174 (8.3)	159 (7.5)	138 (6.9)	146 (7.6)
Low	304 (15)	292 (14)	315 (16)	310 (16)
Medium	457 (22)	488 (23)	496 (25)	436 (23)
High	465 (22)	511 (24)	427 (21)	427 (22)
*N* missing	153 (7.3)	157 (7.4)	177 (8.9)	249 (13)
Body mass index (kg/m^2^) category, *n* (%)				
Underweight	44 (2.1)	46 (2.2)	38 (1.9)	35 (1.8)
Normal weight	650 (31)	638 (30)	561 (28)	490 (25)
Overweight	645 (31)	634 (30)	584 (29)	591 (31)
Obese	721 (35)	791 (37)	792 (40)	782 (41)
*N* missing	26 (1.2)	20 (0.9)	21 (1.1)	32 (1.7)
Diabetes (doctor diagnosed OR HbA_1c_ ≥ 6.5%), *n* (%)				
No	1,711 (82)	1,791 (84)	1,602 (80)	1,548 (80)
Yes	246 (12)	213 (10)	255 (13)	259 (13)
*N* missing	129 (6.2)	125 (5.9)	139 (7.0)	123 (6.4)
History of CVD,^a^ *n* (%)				
No	1,897 (91)	1,947 (91)	1,808 (91)	1,733 (90)
Yes	115 (5.5)	103 (4.8)	126 (6.3)	133 (6.9)
*N* missing	74 (3.5)	79 (3.7)	62 (3.1)	64 (3.3)

CVD, cardiovascular disease; CHD, congenital heart disease; GED, General Educational Development; HbA_1c_, hemoglobin A_1c_; HDL, high‐density lipoprotein; HS, high school; MI, myocardial infarction; NHANES, National Health and Nutrition Examination Survey.

^a^ Self‐reported doctor diagnosed MI, stroke, angina, CHD, or heart failure.

**Table A2:** Detailed Table of Parameters Used in the Model and Methods for Their Estimation

Parameters	Inputs/Mean [Distribution]	Method	Source
Baseline demographic and CVD risk characteristics	Age, gender [M/F], race/ethnicity [White, Black, Hispanic, Asian, Other race], ratio of family income to poverty level, insurance status [Uninsured, Medicare, Medicaid, Private], highest education attained [No degree, GED/HS, Associate/Bachelor degree, Master/Doctorate degree], body mass index (kg/m^2^), total and HDL cholesterol (mg/dL), systolic blood pressure (mm Hg), smoking status (current [Y/N]), hypertension treatment status (current [Y/N]), HbA_1c_ (%), type 2 diabetes status [diagnosed (self‐report) or undiagnosed (no self‐report and HbA_1c_ ≥ 6.5%)], MI, stroke, heart failure, and angina.	—	NHANES 2013/2014
Transition probabilities		
Risk of an MI or stroke	Estimated using age, gender, race/ethnicity, total and HDL cholesterol (mg/dL), systolic blood pressure (mm Hg), smoking status (current [Y/N]), hypertension treatment status, and type 2 diabetes status [diagnosed (self‐report) or undiagnosed (HbA_1c_)]	—	Choi and Basu (2017),^25^ Smith‐Spanlger (2010)^26^
Risk of a fatal MI or stroke	Estimated using age and gender	—	Choi and Basu (2017),^25^ Smith‐Spangler (2010)^26^
Risk of a non‐CVD death	Age‐ and gender‐ specific	—	CDC WONDER^31^
MI or stroke risk multiplier given a history of CVD	2 [Gamma(shape = 3.84, rate = 0.52)]	—	Choi and Basu (2017)^25^
Reduction in systolic blood pressure from receiving Medicaid (mm Hg)	3.03 [Normal(SE = 1.17)]	—	Gotanda et al. (2021)^10^
Reduction in HbA_1c_ from receiving Medicaid (%)^a^	0.14 [Normal (SE = 0.05)]	—	Gotanda et al. (2021)^10^
Costs and utilities		
Utilities (SF‐6D)	QALYs estimated using age category (19‐24, 25–44, 45–64), gender, race/ethnicity, education, family income, insurance status, body mass index, and type 2 diabetes, MI, stroke, heart failure, and angina	—	Morey et al. (2021)^39^
Health care costs (USD 2021)	Annual costs estimated using age category (19‐24, 25–44, 45–64), gender, race/ethnicity, education, family income, insurance status, body mass index, and type 2 diabetes, MI, stroke, heart failure, and angina	—	Morey et al. (2021)^39^
Medicaid costs			
Increase in health care costs in first year of MI or stroke (%)	0.33 [Beta(16.5, 34.3)]	Difference in weighted average MI and stroke health care costs between <1 y ($19,737) and >1 y ($14,894)	Morey et al. (2021)^39^
Increase in medication costs from receiving Medicaid	(223 * (0.21 [Beta(6.9, 25.96)]) + (223 * (0.24 [Beta(11.92, 37.75)])	Per capita US spending on pharmaceuticals in 2014 ($1,122) inflated to 2021 (1,122*1.122 = $1,258) by the proportion of medications related to CVD and diabetes in the United States (14% + 3.7%) equates to $223. This was multiplied by the increase in CVD and diabetes medication costs following Medicaid expansion (21% and 24%)	OECD (2024)^67^; Ghosh et al. (2019)^38^
Increase in routine costs from receiving Medicaid	(1.092 * [186 (Gamma[shape = 25, rate = 0.13])] *[0.02 (Beta[8.03, 414.66])])	Average cost of a routine primary care visit ($186) inflated to 2021 (186*1.092 = 203) by the percentage point increase the proportion of routine visits following Medicaid expansion (2%)	Machlin et al. (2018)^66^; Tummalapalli et al. (2020)^34^
Proportion of which are non‐OOP (%)	1.13 * (increase in health care costs from receiving Medicaid)	Weighted average between US born and immigrant adults (aged 19–64) of change in non‐OOP health care costs ($642) following Medicaid expansion divided by change in total costs ($567)	Kaushal and Muchomba, 2023^46^
Medicaid administration costs (USD 2021)^b^	$250 [Gamma(shape = 25, rate = 0.1)]	Cost per newly eligible adult Medicaid enrollee in 2019 (5,225), Medicaid administration costs as a percentage of total annual costs in 2019 (0.046), inflation index from 2019–2021 (1.04)	Medicaid.gov, 2019^69^; KFF, 2019^44^
Proportion of Medicaid administration costs attributable to CVD (%)	3%	Proportion of Medicaid spending in 2013 on heart disease treatment was estimated at 3%	Renfro, 2018^14^
Lost productivity costs			
Lost productivity from premature mortality (age <65) per year (USD 2021)	$74,000		OECD, 2022^42^
Cost of workplace absenteeism in first year of MI or stroke (USD 2021)	$244 [Gamma(shape = 3.74, rate = 0.015)]	Difference in annual workplace absenteeism costs between matched individuals with ($698/month) and without ($596/month) CVERP; no significant differences after year 1; inflation index from 2013–2021 (1.14)	Song et al., 2015^41^
Cost of short‐term disability claims in first year of MI or stroke (USD 2021)	$897 [Gamma(shape = 328.88, rate = 0.37)]	Difference in short‐term disability claims between matched individuals with ($281/month) and without ($60/month) CVERP; no significant differences after year 1; inflation index from 2013–2021 (1.14)	Song et al., 2015^41^

CDC, Centers for Disease Control and Prevention; CVD, cardiovascular disease; CVERP, cardiovascular events and related clinical procedures; F, female; GED, general education development; HbA_1c_, hemoglobin A_1c_; HDL, high‐density lipoprotein; HS, high school; KFF, Kaiser Family Foundation; M, male; MI, myocardial infarction; N, no; NHANES, National Health and Nutrition Examination Survey; OCED, Organisation of Economic Co‐operation and Development; QALY, quality adjusted life‐year; SE, standard error; SF‐6D, short‐form 6 dimension; USD, United States dollars; OOP, out‐of‐pocket; Y, yes.

^a^ We assumed that this reduction in HbA_1c_ only occurred for those without a diagnosis of diabetes.

^b^ Because no measure of dispersion was available for this estimate, an SE was estimated as 20% of the mean value (250 × 0.2) and used to estimate the shape and rate for a gamma distribution.

**Table A3:** Incidence Rates Per 100,000 Person‐Years for the Base‐Case Scenario (Lifetime Horizon [or Age 85], Whole Population Aged 19–64, Societal Perspective) in Total and According to Age and Equity‐Relevant Groups

Category	Arm	IR MI	MCSE	IR Stroke	MCSE	IR CVD Death	MCSE	IR Non‐CVD Death	MCSE	*n*
Total	nME	814.9	5.0	636.6	4.3	250.9	1.7	2,831.8	2.4	162,820,000
	ME	804.2	4.9	628.5	4.2	247.2	1.7	2,832.3	2.3	
Difference		−10.7	1.0	−8.1	0.9	−3.6	0.7	0.5	1.6	
Medicaid eligible	nME	952.9	9.3	686.4	7.2	284.0	3.8	2,421.7	12.1	16,916,248
	ME	848.4	10.0	609.7	7.2	249.6	3.7	2,429.7	10.4	
Difference		−104.5	4.1	−76.7	4.4	−34.3	1.9	8.0	6.1	
Age category										
<25	nME	575.9	3.7	401.6	3.0	136.2	1.2	1,383.0	3.6	21,866,853
	ME	570.4	3.6	397.1	3.0	134.7	1.2	1,383.3	4.0	
Difference		−5.6	1.3	−4.5	1.1	−1.6	0.8	0.3	2.6	
										
25‐44	nME	704.9	4.1	513.2	3.3	189.1	1.4	1,943.3	2.8	7,010,4946
	ME	698.9	4.1	509.2	2.9	187.5	1.4	1,944.0	2.9	
Difference		−6.1	1.2	−4.1	1.0	−1.6	0.8	0.7	2.1	
45‐64	nME	997.5	6.4	831.2	5.9	347.4	2.5	4,158.0	5.2	70,848,201
	ME	980.6	6.3	818.0	5.9	341.1	2.5	4,158.4	5.4	
Difference		−16.8	2.2	−13.2	1.7	−6.3	1.3	0.4	3.8	
Highest educational achievement										
Associate/Bachelor	nME	813.3	5.3	646.7	4.4	243.5	2.2	2,731.7	7.7	53,158,100
	ME	809.5	5.5	643.3	4.2	242.3	1.9	2,732.3	7.5	
Difference		−3.8	1.6	−3.4	1.3	−1.2	1.3	0.6	4.2	
GED/HS	nME	960.8	6.5	735.6	5.9	301.2	2.2	2,902.8	8.6	365,39209
	ME	929.5	6.0	713.8	5.5	289.7	2.3	2,905.2	9.0	
Difference		−31.3	2.6	−21.8	2.6	−11.5	1.7	2.4	3.5	
Master/Doctorate	nME	626.3	4.4	498.7	3.9	194.4	1.8	2,876.2	7.1	49,959,164
	ME	626.2	4.3	497.8	3.8	194.5	1.9	2,875.6	6.1	
Difference		−0.2	1.2	−0.9	1.3	0.1	1.1	−0.6	3.5	
No degree	nME	995.0	8.3	754.6	5.7	310.0	3.5	2,853.3	9.0	23,163,527
	ME	978.4	7.5	741.6	5.3	305.1	2.6	2,853.0	7.9	
Difference		−16.6	2.4	−13.0	2.3	−4.9	2.1	−0.3	5.2	
Family income										
High	nME	699.9	5.2	556.4	4.2	226.7	2.5	3,184.0	7.2	55,048,838
	ME	700.4	5.0	556.3	4.0	226.6	2.4	3,183.5	6.9	
Difference		0.5	1.3	−0.1	1.3	−0.1	1.1	−0.5	3.4	
Low	nME	876.2	10.7	668.2	7.8	263.3	3.7	2,643.3	10.7	23,788,854
	ME	868.8	10.0	660.8	7.1	261.0	3.9	2,642.1	11.8	
Difference		−7.4	3.0	−7.4	3.3	−2.3	1.4	−1.2	4.3	
Medium	nME	819.6	7.8	651.2	6.4	253.7	3.0	2,815.9	11.0	45,043,162
	ME	819.6	7.4	650.9	6.6	253.8	2.9	2,815.2	11.4	
Difference		0.0	1.5	−0.2	1.5	0.1	1.0	−0.7	3.4	
Near poor	nME	891.2	14.0	675.1	9.5	263.2	5.2	2,514.2	17.7	10,197,621
	ME	871.9	14.4	660.4	9.3	257.6	5.3	2,515.9	18.5	
Difference		−19.3	4.2	−14.7	2.7	−5.6	3.4	1.6	9.5	
										
Poor	nME	949.9	9.3	727.6	7.7	278.1	3.1	2,450.7	5.7	28,741,525
	ME	901.4	9.5	693.5	6.8	261.3	3.1	2,455.9	5.6	
Difference		−48.5	2.6	−34.0	2.3	−16.8	1.5	5.3	3.9	
Race/ethnicity										
Asian	nME	715.0	6.2	568.0	4.0	220.3	3.4	2,681.7	11.3	9,477,426
	ME	712.6	5.3	566.5	5.9	219.6	2.5	2,679.6	10.8	
Difference		−2.4	4.0	−1.5	3.9	−0.7	2.3	−2.1	7.6	
Black	nME	1,017.0	7.3	754.5	6.6	301.0	3.1	2,677.3	6.2	19,980,531
	ME	1,004.4	7.7	742.7	5.9	297.7	2.9	2,677.9	6.1	
Difference		−12.7	3.4	−11.8	3.1	−3.4	1.8	0.6	4.8	
Hispanic	nME	819.5	5.3	627.5	4.6	246.7	2.1	2,518.0	7.1	27,852,953
	ME	807.4	4.7	618.7	4.8	243.3	2.1	2,519.2	6.4	
Difference		−12.1	2.1	−8.9	1.8	−3.3	1.6	1.3	4.0	
Other race	nME	840.9	7.9	674.8	6.6	250.1	3.3	2,628.6	14.5	5,872,830
	ME	835.9	8.2	672.2	6.5	248.6	3.5	2,628.5	12.2	
Difference		−5.0	4.8	−2.5	5.5	−1.5	3.4	−0.1	9.7	
White	nME	781.0	4.7	619.7	4.1	244.9	1.7	2,976.7	3.6	99,636,260
	ME	770.0	4.6	611.6	3.9	240.7	1.7	2,977.2	3.5	
Difference		−11.0	1.3	−8.1	1.4	−4.2	0.9	0.5	2.7	

CVD, cardiovascular disease; GED, general educational development; HS, high school; IR, incident rate; ME, Medicaid expansion; MI, myocardial infarction; MSCE, Monte Carlo standard error; nME, no Medicaid expansion.

**Table A4 milq70004-tbl-0005:** Incidence Rates Per 100,000 Person‐Years Examining for Different Lengths of Follow‐Up in Total and Across Age‐Groups for the ME Arm

		MI	MCSE	Stroke	MCSE	CVD Death	MCSE	Non‐CVD Death	MCSE
Total	1‐y follow‐up	167.8	1.4	80.9	0.7	0.0	0.0	396.0	2.0
	10‐y follow‐up	401.4	2.1	230.3	1.2	82.4	0.6	1,211.6	2.2
	Lifetime or age 85	804.2	4.9	628.5	4.2	247.2	1.7	2,832.3	2.3
Age‐group									
<25	1‐y follow‐up	71.8	2.1	12.2	0.6	0.0	0.0	79.0	3.8
	10‐y follow‐up	155.8	1.3	40.1	0.6	15.3	0.6	255.1	3.9
	Lifetime or age 85	570.4	3.6	397.1	3.0	134.7	1.2	1,383.3	4.0
25‐44	1‐y follow‐up	119.8	1.3	35.6	0.9	0.0	0.0	149.7	2.1
	10‐y follow‐up	264.6	1.5	102.3	0.6	37.4	0.6	482.0	2.8
	Lifetime or age 85	698.9	4.1	509.2	2.9	187.5	1.4	1,944.0	2.9
45‐64	1‐y follow‐up	244.8	2.8	147.0	1.6	0.0	0.0	737.6	4.9
	10‐y follow‐up	612.6	3.7	415.6	2.5	147.7	1.3	2,228.6	5.3
	Lifetime or age 85	980.6	6.3	818.0	5.9	341.1	2.5	4,158.4	5.4

**Table A5:** Cost‐effectiveness results for the base‐case scenario in total and across equity‐relevant groups

Category	Arm	Costs	MCSE	QALYs	MCSE	NHB	ICER	INHB	MCSE	*n*
Total	nME	270,551	1,453	15.69077	0.01042	13.88709				
	ME	27,0525	1,454	15.69367	0.01059	13.89017	−8,931	0.00307	0.00147	162,820,000
Medicaid eligible only	nME	206,072	1,096	15.94806	0.01706	14.57425				
	ME	203,926	1,125	15.97605	0.01772	14.61654	−76,679	0.0423	0.00708	16,916,248
Age category										
<25	nME	269,328	1,309	19.94709	0.01423	18.15156				
	ME	269,343	1,301	19.94835	0.01434	18.15273	11,896	0.00117	0.00471	21,866,853
25‐44	nME	286,115	1,429	17.63251	0.01135	15.72508				
	ME	286,181	1,431	17.63365	0.01164	15.72577	58,252	7e‐04	0.00242	70,104,946
45‐64	nME	255,528	1,567	12.45554	0.00853	10.75202				
	ME	255,398	1,573	12.4607	0.00855	10.75805	−25,163	0.00603	0.00178	70,848,201
Highest educational achievement										
Associate/ Bachelor	nME	277,651	1,652	15.91038	0.01784	14.05937				
	ME	277,755	1,668	15.91139	0.01781	14.05969	102,598	0.00032	0.00319	53,158,100
GED/HS	nME	261,510	1,498	15.25703	0.02187	13.51363				
	ME	261,225	1,470	15.26432	0.02152	13.52281	−39,038	0.00918	0.00311	36,539,209
Master/Doctorate	nME	284,426	1,663	16.19157	0.014	14.2954				
	ME	284,683	1,657	16.19139	0.01391	14.29351	−1,408,574	−0.0019	0.00262	49,959,164
No degree	nME	238,593	1,182	14.79076	0.02063	13.20015				
	ME	238,067	1,157	14.79773	0.02145	13.21062	−75,547	0.01048	0.00508	2,316,3527
Family income										
High	nME	274,874	1,563	15.66445	0.01668	13.83196				
	ME	275,157	1,561	15.6646	0.01656	13.83022	1,903,874	−0.00174	0.00252	55,048,838
Low	nME	276,272	1,716	15.72665	0.02832	13.88484				
	ME	275,964	1,724	15.73047	0.02872	13.89071	−80,611	0.00587	0.00417	23,788,854
Medium	nME	263,621	1,592	15.8728	0.01982	14.11532				
	ME	263,915	1,563	15.8729	0.02021	14.11347	2,874,219	−0.00186	0.00269	45,043,162
Near Poor	nME	264,494	1,530	15.53914	0.02781	13.77585				
	ME	263,950	1,553	15.54538	0.02826	13.78571	−87,109	0.00987	0.00652	10,197,621
Poor	nME	270,547	1,456	15.47999	0.0134	13.67634				
	ME	269,846	1,486	15.4906	0.01338	13.69163	−66,050	0.01529	0.00376	28,741,525
Race/Ethnicity										
Asian	nME	254,169	1,498	16.4784	0.01074	14.78394				
	ME	254,364	1,578	16.47993	0.01085	14.78417	127,590	0.00023	0.00691	9,477,426
Black	nME	274,306	1,466	15.68303	0.01246	13.85433				
	ME	274,072	1,495	15.68517	0.01344	13.85802	−109,603	0.00369	0.00516	19,980,531
Hispanic	nME	234,683	1,161	16.32091	0.00948	14.75635				
	ME	234,543	1,152	16.32459	0.01062	14.76097	−38,124	0.00462	0.00409	27,852,953
Other race	nME	302,543	2,118	15.65214	0.01509	13.63518				
	ME	302,644	2,053	15.65364	0.0168	13.63602	67,113	0.00083	0.00861	5,872,830
White	nME	279,497	1,590	15.44354	0.0115	13.58023				
	ME	279,516	1,587	15.44659	0.01114	13.58315	6,356	0.00292	0.00208	99,636,260

CVD, cardiovascular disease; ICER, incremental cost‐effective ratio; INHB, incremental net health benefit; ME, Medicaid expansion; MI, myocardial infarction; MSCE, Monte Carlo standard error; NHB, net health benefit; nME, no Medicaid expansion; QALY, quality adjusted life‐year.

### Lost Productivity

Although a systematic review was identified which examined the productivity losses associated with CVD,^62^ we opted for an estimate from a single study, published after the search date of this review but with a more robust study design than studies included in the review.^41^ Song and colleagues (2015) matched a cohort of individuals with and without cardiovascular events and related clinical procedures (CVERP) and compared workplace absenteeism and short‐term disability costs associated with cardiovascular disease in the United States.^41^ Costs were estimated on a monthly basis in 2013 USD and were therefore converted to annual costs by multiplying by 12 and to 2021 USD by using the overall personal consumption expenditure price index^45^ (1.14).

### Medicaid Administration Cost

Medicaid spending per adult enrollee newly eligible as part of the ACA Medicaid expansion was estimated to be $5,225 in 2019 across the United States.^44^ For the same year (2019), total Medicaid spending was estimated to be $646 billion, of which $30 billion was for Medicaid administration (30/646 = 0.05). We estimated the administration cost per enrollee as 0.05*$5,225 and inflated this to 2021 prices using the personal consumption expenditure price index for health^45^ (1.04). The proportion of Medicaid expenditure attributed to heart disease was 3%.^14^

### Health Care Costs and QALYs

These were estimated for each individual in the model annually using the equation from Morey and colleagues (2021),^39^ which predicted the probability of having nonzero medical expenditures and the cost of nonzero expenditures annually, as well as an individual's annual utility using the SF‐6D.^40^ NHANES variables were formatted to match those variables used in the Morey and colleagues (2021) equation:

1. Age was continuous in NHANES and was categorized into four groups: 18 to 24; 24 to 44; 45 to 64; 65+.
2. Family income was grouped into five categories: poor (income ≤100% of the FPL); near poor (100% < income ≤ 125%); low (125% < income ≤ 200%); medium (200% < income ≤ 400%); high (400% < income)
3. Insurance status roughly matched those from NHANES (Private, Medicaid, Medicare, other plan, and uninsured), however as Morey and colleagues (2021) did not include a variable for “other plan”, and the majority of publicly funded plans for ages 19 to 64 are Medicaid, we used the Medicaid coefficient to predict their baseline costs.
4. Highest educational achievement was recategorized from the five levels in NHANES to four levels: No degree, GED/HS, Associate/Bachelor degree, or Master/Doctorate degree.
5. Continuous BMI was split into four categories: Underweight (BMI < 18.5kg/m^2^); Normal weight (18.5 ≤ BMI < 25); Overweight (25 ≤ BMI < 30); and Obese (30 ≥ BMI).

Other variables included in the prediction equation which aligned with NHANES data were MI, stroke, diabetes, angina, heart failure, sex, and race. Where data for the prediction equation were not available in NHANES, a constant value was used for all individuals: cardiac dysrhythmia = zero; peripheral artery disease = zero; and Charlson Comorbidity Score = one. Health care costs were inflated from 2017 to 2021 prices using the personal consumption expenditure price index for health^45^ (1.07).

### Effect of Medicaid on Health Care Costs

Some studies have compared differences in medical expenditures between uninsured and Medicaid individuals, estimating expenditures to be approximately $5,000 higher among those with Medicaid.^39^ ^,63,64^ However, these are subject to a number of biases, for example newly eligible Medicaid enrollees were found to have lower health care utilization and costs, than those already enrolled^65^ suggesting differences in the underlying health of enrolling individuals as eligibility expands. Yet, it is likely that receiving Medicaid increased health care costs as individuals’ health care utilization for preventive services increased.^13^, ^37^

To model the total increase in health care costs from preventive services related to CVD,^13^ we estimate an increase in the cost of routine primary care visits as the average cost of a visit ($186)^66^ by the percentage point increase in the proportion of visits following expansion (2%).^34^ The increase in medication costs was estimated as the share of the 2014 average per capita pharmaceutical spending in the United States ($1,122)^67^ related to cardiovascular and diabetes medications (14% and 4%)^38^ by the proportionate increase in CVD and diabetes medication use following expansion (21% and 24%).^38^ This was inflated to 2021 USD (1.12).^68^

To consider the proportion of this total increase in health care costs which accrue to the individuals receiving Medicaid, we estimated the proportion of the weighted average (between US and non‐US born populations) of non‐OOP costs ($643) to the weighted average of total costs ($568) to be 1.13.^2^ Thus, we assumed that 113% of the increase in total costs per individual per year were incurred as Medicaid costs, reflecting a 13% reduction in OOP costs per person. The allocation of the increase in non‐OOP costs is discussed in the main text.

### CVD History

Individuals were divided into states at the beginning of the model according to those with and without a history of CVD (Figure 1). This was defined as “CVD history” if individuals reported doctor diagnosed MI, Stroke, angina, coronary heart disease or heart failure at baseline and “No CVD history” otherwise. Annual risk of MI or stroke (calculated below) was multiplied by a factor of two (Table A2) for those with a history of CVD.^25^

### Non‐CVD Mortality

This was estimated by taking 2013 to 2014 death rates (with SEs) from the CDC WONDER database by individual age and gender for all‐cause underlying cause of death (rather than multiple causes of death) and separately for acute MI (ICD‐10 codes I21‐22^32^) and stroke/cerebrovascular disease (ICD‐10 codes I60‐I69^33^). To estimate the risk of a non‐CVD death, specifically non‐MI and nonstroke death, we subtracted the annual death rates with each age and gender for MI and stroke from all‐cause deaths and converted them to annual probabilities.^21^ These were available for ages 18 to 84. In some cases, estimates were unavailable, owing to small numbers, for those aged under 25 years (e.g., stroke death rates for females aged 19 years) and so we used the five‐years age‐group (15‐19 and 20–24) death rates in these cases.

### Risk of Myocardial Infarction (MI) or Stroke

To estimate the risk of an MI or stroke we follow the approach by Choi and Basu (2017) who used validated equations of monthly risk of MI and stroke according to age and sex.^25^ We estimated the monthly risk per person using this approach and converted to annual probabilities assuming a constant rate over time.^21^ The ASCVD equation^27^ reflects greater variation in risk according to race, sex, age, total and HDL cholesterol, systolic blood pressure, hypertension treatment status, smoking status, and diabetes status. However, the ASCVD risk equation does not differentiate between fatal and nonfatal MI or stroke, which are important as separate outcomes for cost‐effectiveness models. Choi and Basu (2017) use an approach which weights the monthly risk of MI or stroke by the individual to average ASCVD risk within groups according to age, sex, race, and income. We found this to be unstable as reductions in systolic blood pressure and HbA_1c_ from receiving Medicaid^10^ could alter both the denominator and numerator of the weights thereby unrealistically increasing risk for some individuals even if their blood pressure and HbA_1c_ was reduced.

As such we used a similar approach which estimated MI (or stroke) risk, as described above, according to age and sex for everyone at baseline (part 1 below). We then estimated ASCVD risk at baseline using the ASCVD risk equation and averaged risk over age and sex (part 2) to estimate the probability of having an MI given an individual had had an ASCVD event (part 3). Within each time cycle (1 year) of the model we then estimated an individual's ASCVD risk (part 4) and multiplied this by their baseline probability that their ASCVD event would be an MI (part 3) to estimate with greater granularity their annual probability of an MI (part 5) (i.e., according to ASCVD risk equation variables). This also allowed us to model the reduction in MI risk because of intervention on systolic blood pressure and HbA_1c_. This process was repeated to estimate annual stroke risk.

### Steps for using Bayes formulation combine ASCVD equation characteristics with MI (or stroke) risk equations

1. Estimate pr(MI) by age group and sex; pr(MI | age group, sex)
2. Estimate pr(ASCVD) and average over age group and sex; pr(ASCVD | age group, sex)
3. Estimate pr(MI | ASCVD) = pr(MI | age group, sex) / pr(ASCVD | age group, sex)
4. Estimate pr(ASCVD) by all characteristics; pr(ASCVD | all)
5. Estimate pr(ASCVD, MI) = pr(MI | ASCVD) * pr(ASCVD)

Steps 1–3 were fixed across arms while Steps 4–5 varied across arms.

## Results

### Risk of MI and Stroke

Incidence rates per 100,000 person‐years for the base‐case scenario are provided in Table A3 below in total and according to age‐groups and equity‐relevant variables (family income, education and race/ethnicity). The analysis shows that incidence of MI was 805 and stroke was 630 per 100,000 person‐years in the expansion arm. This risk was higher among older age‐groups and was lower when we ran the model for a shorter time periods (Table A4).

### Validation Stroke

Petrea and colleagues (2009) estimated the US incidence of stroke for females aged 45 years at baseline to range from 0.79 to 13.85 per 1,000 person‐years depending on the time horizon (10 to 40 year follow‐up) and 1.08 to 14.23 for males.^70^ The corresponding incidence rates for those aged 55 at baseline were 1.7 to 19.39 for females and 2.42 to 15.97 for males. Madsen and colleagues (2020) estimated age‐specific US incidence rates for a one‐year period in 2015 to be 0.28 per 1,000 person‐years for those aged 20 to 44 at baseline and 1.71 for those aged 45 to 64.^71^ Our results for all individuals aged 45 to 64 were 1.5, 4.2 and 8.2 per 1,000 person‐years for the one‐year, ten‐year, and lifetime follow‐up periods and 0.4, 1.0 and 5.1 for those aged 25 to 44 suggesting our estimates of the rate of stroke are consistent with other US estimates. We also observe an increasing incidence rate as the time period of follow‐up increases.

### Validation MI

Parikh and colleagues (2009) estimated the incidence of MI in the United States over a ten‐year period (1990‐1999) to be 2.47, 3.98 and 5.11 per 1,000 person‐years for males aged 40 to 49, 50 to 59, and 60 to 69, respectively, and 1.6, 2.17, and three for females according to the same age‐groups.^72^ Yeh and colleagues (2010) also estimated the age‐adjusted incidence of MI in the United States in 2008 over a one‐year period to be 2.1 per 1,000 person‐years. Our estimates of the incidence rates for the one‐year and ten‐year time horizons were 1.2 and 2.7 for all individuals aged 25 to 44 at baseline, and 2.7 and 6.1 for those aged 45 to 64. Over the lifetime horizon (or age 85 years) this increased to 7 and 9.8 per 1,000 person‐years for the corresponding age‐groups. Although definitions of MI (or stroke) and follow‐up periods may vary across studies, our estimates of MI and stroke were consistent with published sources.

### CVD Deaths

Of note, our estimate of the reduction in CVD mortality from Medicaid expansion (3.6 per 100,000 person‐years; Table A3) is consistent with estimates which were not used to parameterize this model; Khatana and colleagues estimated a reduction in CVD mortality of 4.3 (95% CI, 1.8‐6.9) following Medicaid expansion.^8^ [Table milq70004-tbl-0005]

**Appendix References**

61. Selvin E, Wang D, Lee AK, Bergenstal RM, Coresh J. Identifying trends in undiagnosed diabetes in U.S. adults by using a confirmatory definition: a cross‐sectional study. *Ann Intern Med*. 2017;167(11):769‐776. https://doi.org/10.7326/M17‐1272

62. Gordois AL, Toth PP, Quek RG, Proudfoot EM, Paoli CJ, Gandra SR. Productivity losses associated with cardiovascular disease: a systematic review. *Expert Rev Pharmacoecon Outcomes Res*. 2016;16(6):759‐769. https://doi.org/10.1080/14737167.2016.1259571

63. Zhang D, Ritchey MR, Park C, Li J, Chapel J, Wang G. Association between Medicaid coverage and income status on health care use and costs among hypertensive adults after enactment of the Affordable Care Act. *Am J Hypertens*. 2019;32(10):1030‐1038. https://doi.org/10.1093/ajh/hpz101

64. Guth M, Garfield R, Rudowitz R. The effects of Medicaid expansion under the ACA: studies from January 2014 to January 2020. Kaiser Family Foundation (KFF). 2020. https://www.kff.org/medicaid/report/the‐effects‐of‐medicaid‐expansion‐under‐the‐aca‐updated‐findings‐from‐a‐literature‐review/

65. Jacobs PD, Kenney GM, Selden TM. Newly eligible enrollees in Medicaid spend less and use less care than those previously eligible. *Health Aff (Millwood)*. 2017;36(9):1637‐1642. https://doi.org/10.1377/hlthaff.2017.0252

66. Machlin SR, Mitchell EM. Statistical brief #517: expenses for office‐based physician Vvsits by specialty and insurance type, 2016. 2018. Accessed October 29, 2024. https://meps.ahrq.gov/mepsweb/data_files/publications/st517/stat517.shtml

67. Organisation of Economic Co‐operation and Development (OECD). Pharmaceutical spending: indicator. 2024. Accessed October 30, 2024. https://www.oecd.org/en/data/indicators/pharmaceutical‐spending.html

68. Dunn A, Grosse SD, Zuvekas SH. Adjusting health expenditures for inflation: a review of measures for health services research in the United States. *Health Serv Res*. 2018;53(1):175‐196. https://doi.org/10.1111/1475‐6773.12612

69. Medicaid.gov. Annual Medicaid & CHIP expenditures. 2024. Accessed May 16, 2024. https://www.medicaid.gov/state‐overviews/scorecard/main

70. Petrea RE, Beiser AS, Seshadri S, Kelly‐Hayes M, Kase CS, Wolf PA. Gender differences in stroke incidence and poststroke disability in the Framingham heart study. *Stroke*. 2009;40(4):1032‐1037. https://doi.org/10.1161/STROKEAHA.108.542894

71. Madsen TE, Khoury JC, Leppert M, et al. Temporal trends in stroke incidence over time by sex and age in the GCNKSS. *Stroke*. 2020;51(4):1070‐1076. https://doi.org/10.1161/STROKEAHA.120.028910

72. Parikh NI, Gona P, Larson MG, et al. Long‐term trends in myocardial infarction incidence and case fatality in the National Heart, Lung, and Blood Institute's Framingham heart study. *Circulation*. 2009;119(9):1203‐1210. https://doi.org/10.1161/CIRCULATIONAHA.108.825364
